# Supplementary figures and images for: Characterisation of the R2R3 Myb subgroup 9 family of transcription factors in tomato
Source: PLoS One. 2024 Mar 26;19(3):e0295445. doi: 10.1371/journal.pone.0295445 (PMC10965086; doi:10.1371/journal.pone.0295445)

A

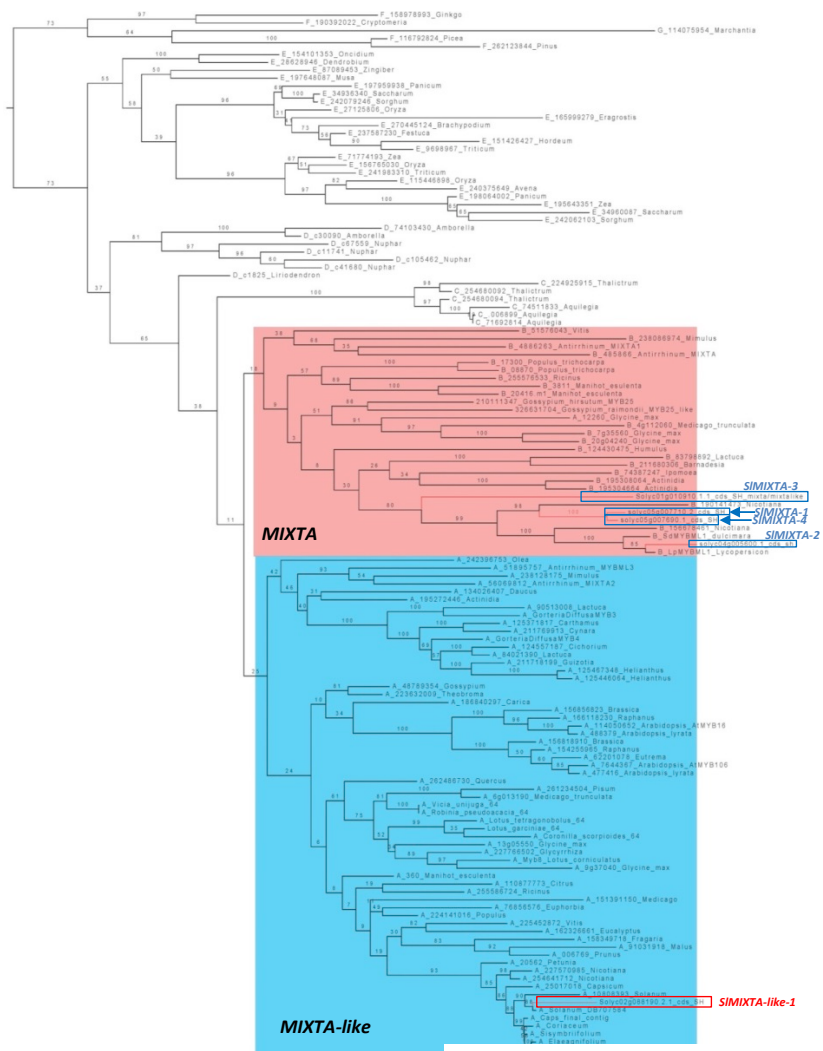

B

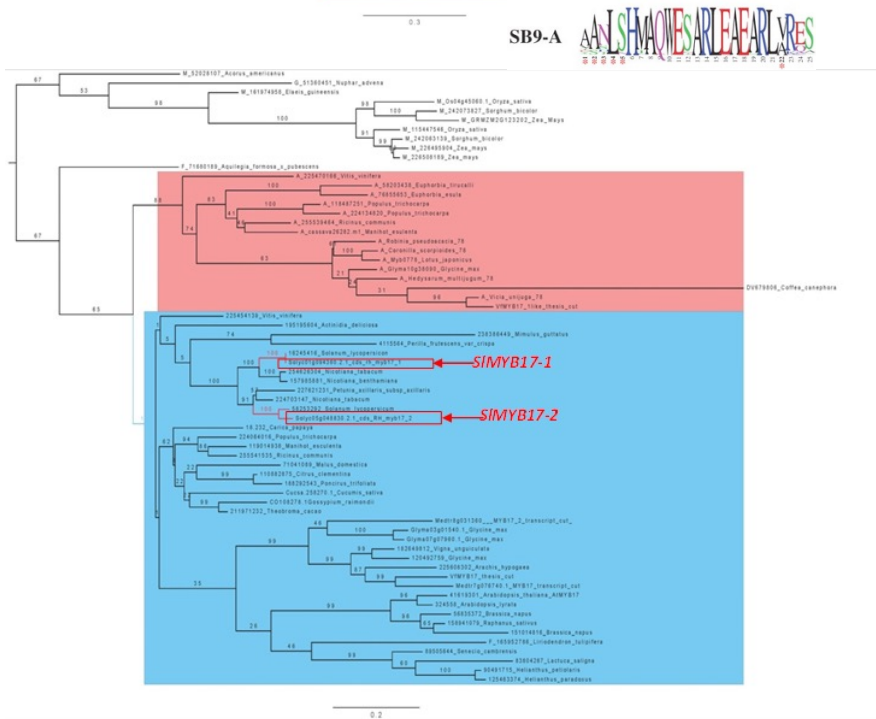

Supplement: S1 Fig — S1A shows subgroup 9A. S1B shows subgroup 9B. (PDF) [file pone.0295445.s001.pdf]

*Sl-MIXTA-1*

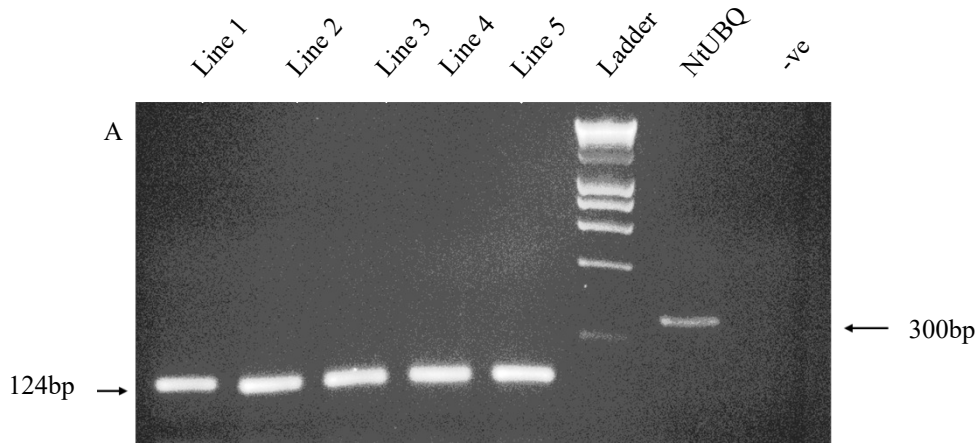

*Sl-MIXTA-2*

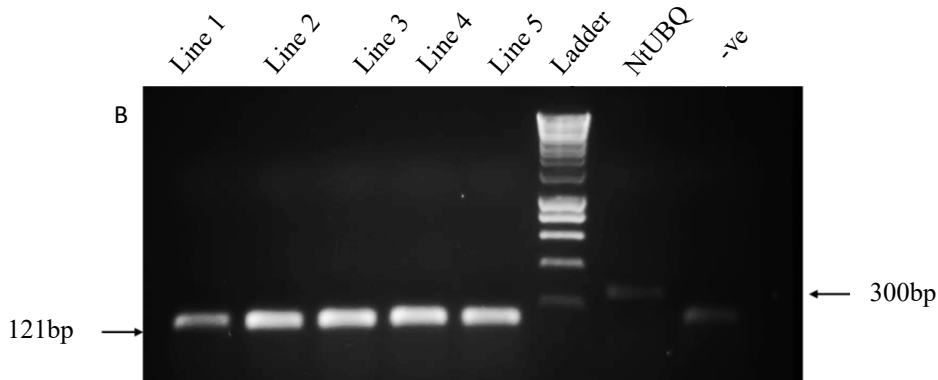

*Sl-MIXTA-3*

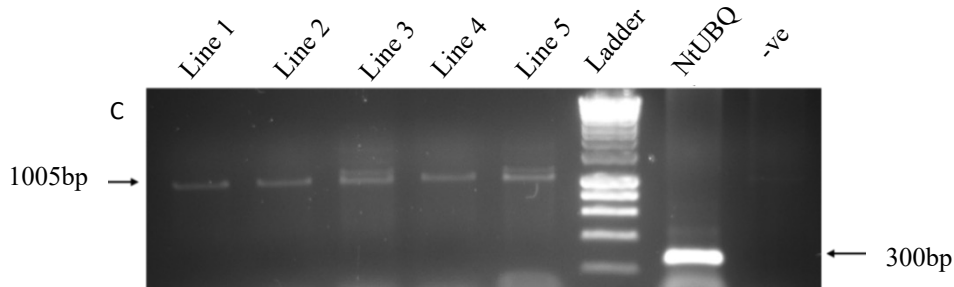

*Sl-MIXTA-4*

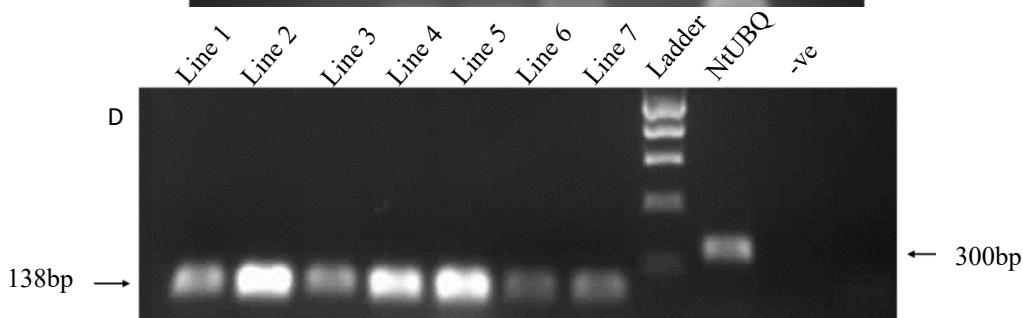

*Sl-MIXTA-like-1*

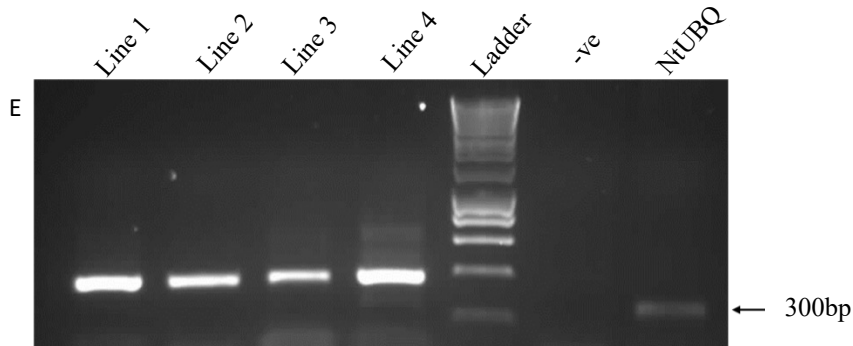

Supplement: S2 Fig — (PDF) [file pone.0295445.s002.pdf]

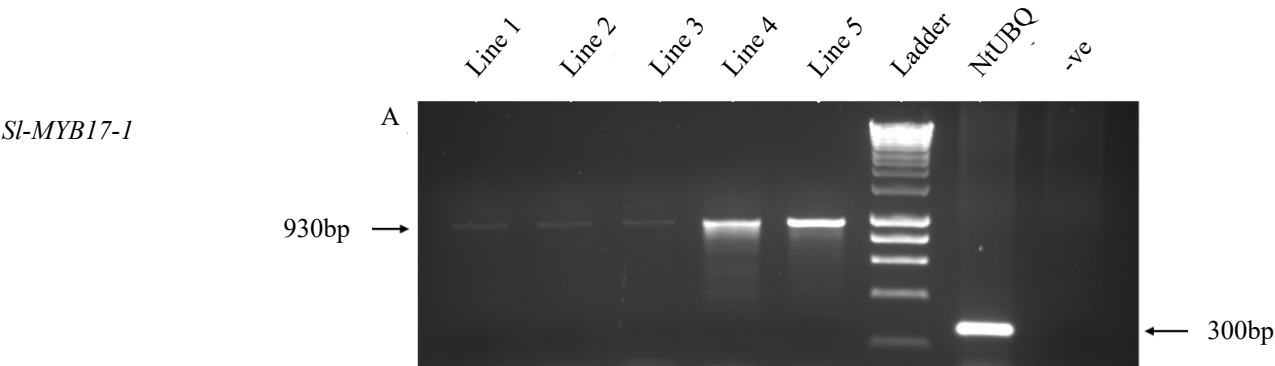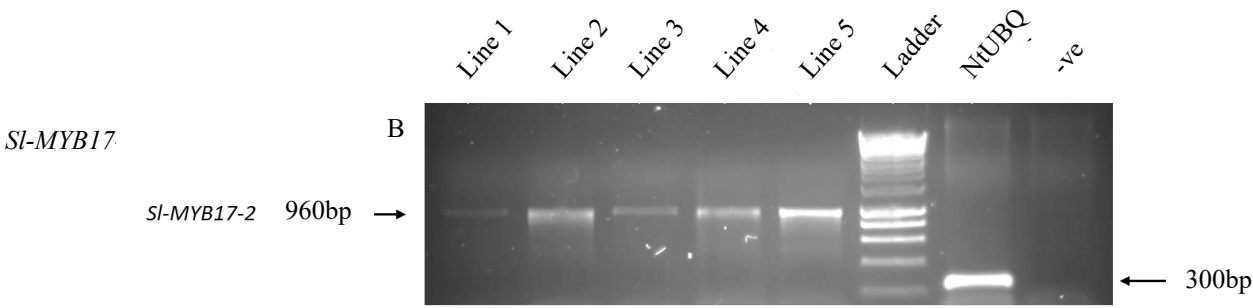

Supplement: S3 Fig — (PDF) [file pone.0295445.s003.pdf]
