# Supplementary material for: Characterisation of the R2R3 Myb subgroup 9 family of transcription factors in tomato
Source: PLoS One. 2024 Mar 26;19(3):e0295445. doi: 10.1371/journal.pone.0295445 (PMC10965086; doi:10.1371/journal.pone.0295445)

S4

SlMYB17-1 semiQPCR uncropped Gel image used for Fig.5

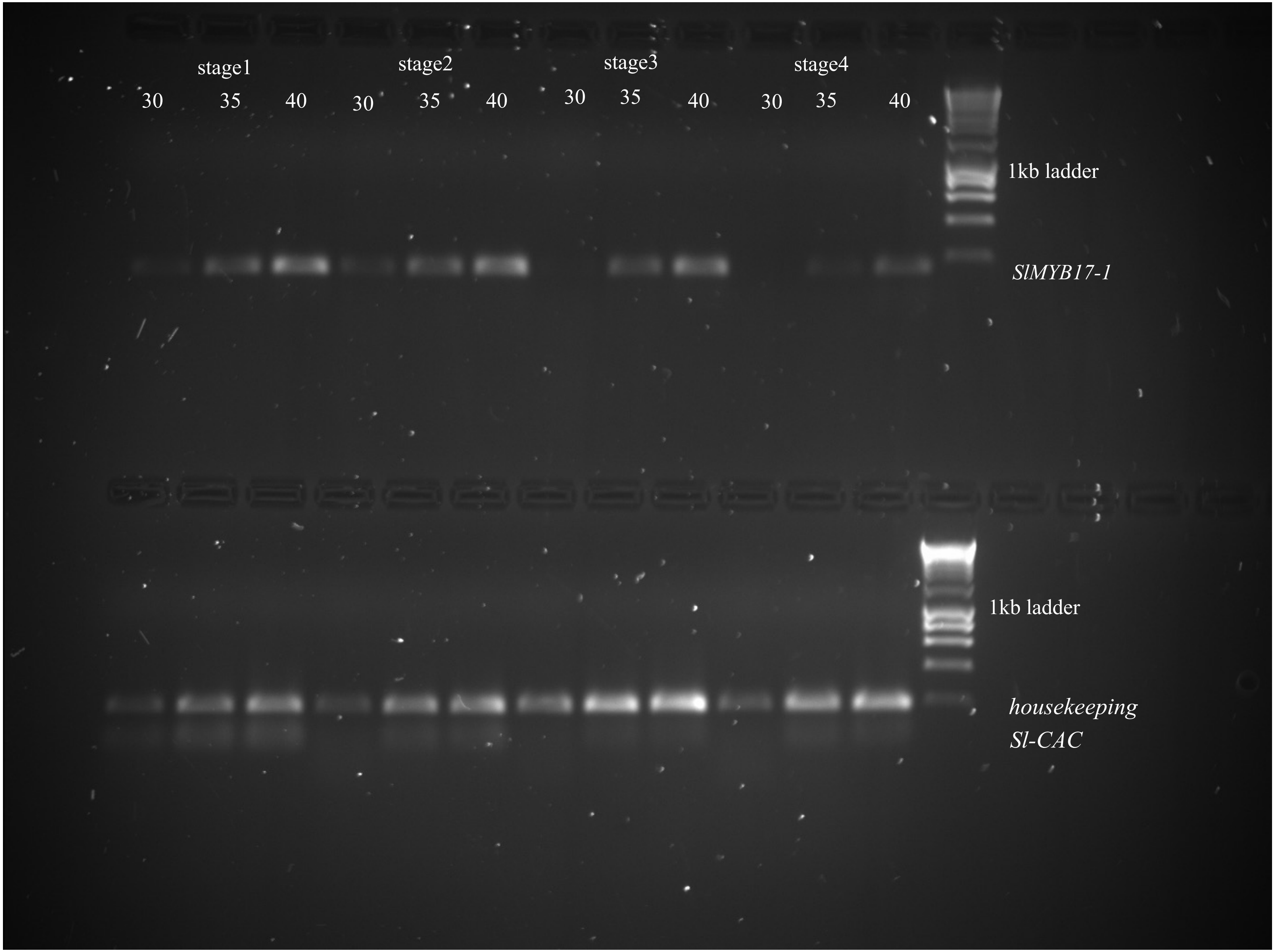

SlMYB17-2 semiQPCR uncropped Gel image used for Fig.5

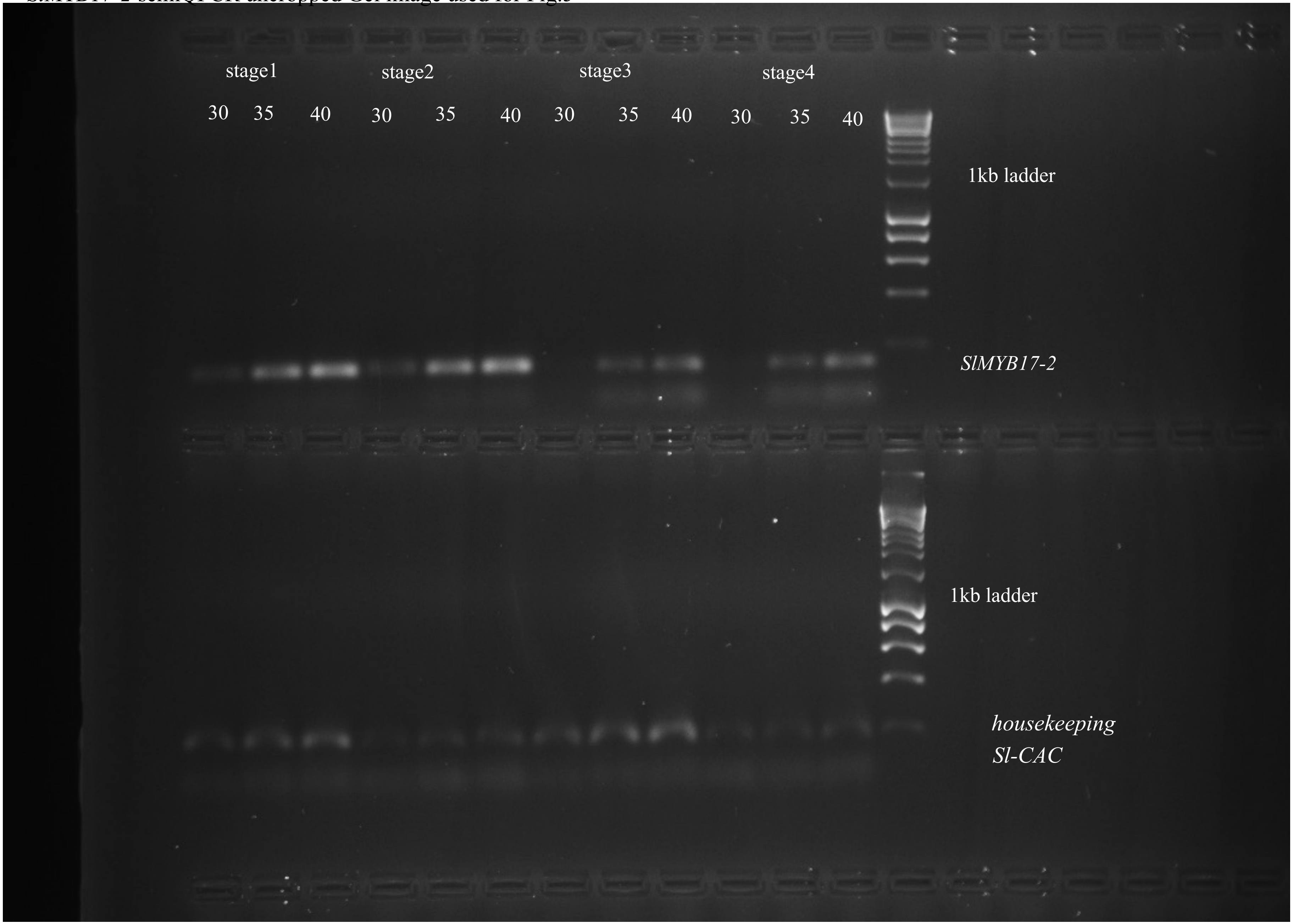

SlMIXTA-like-1 semiQPCR uncropped Gel image used for Fig.5

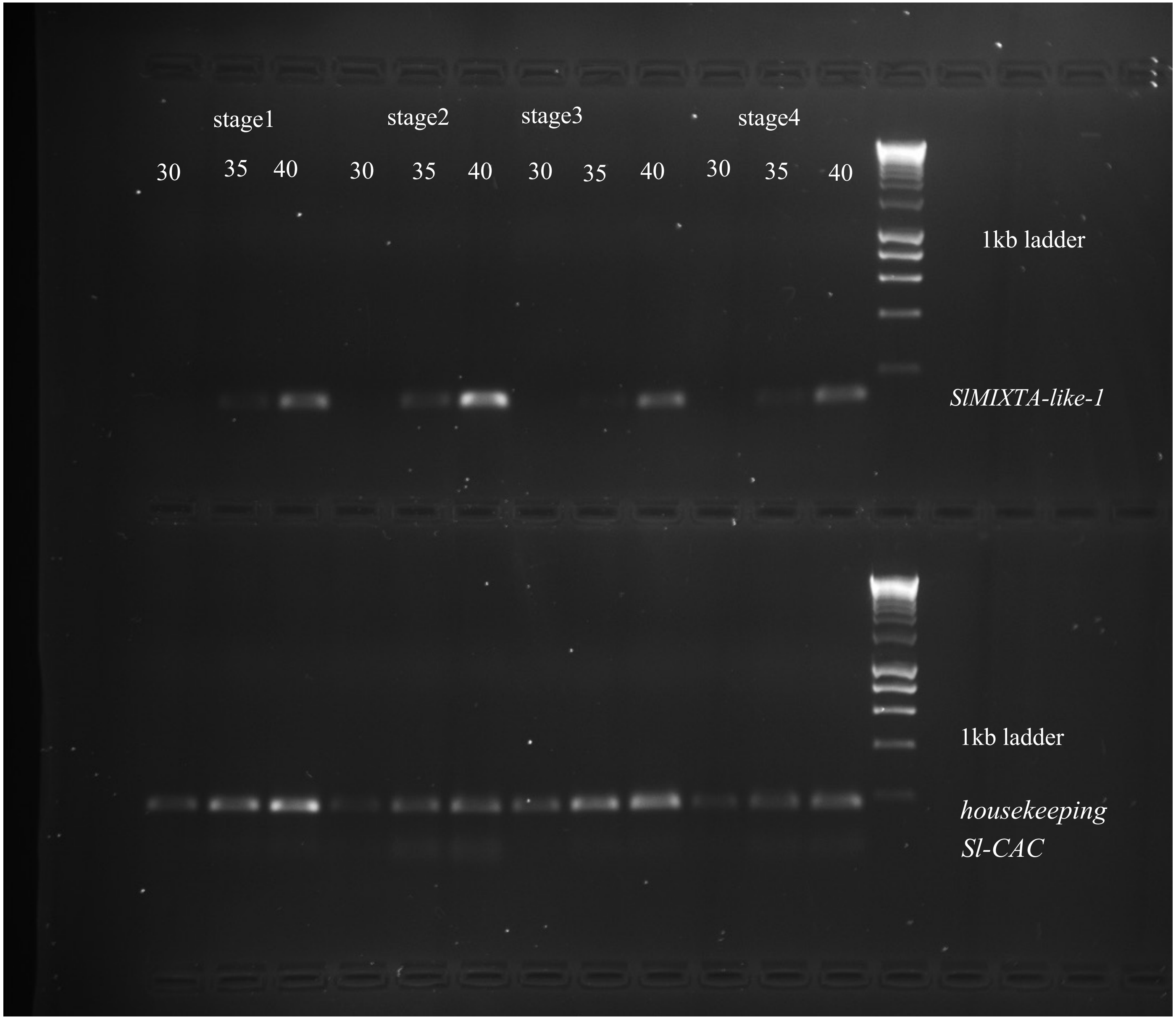

SlMIXTA-1 semiQPCR uncropped Gel image used for Fig.5

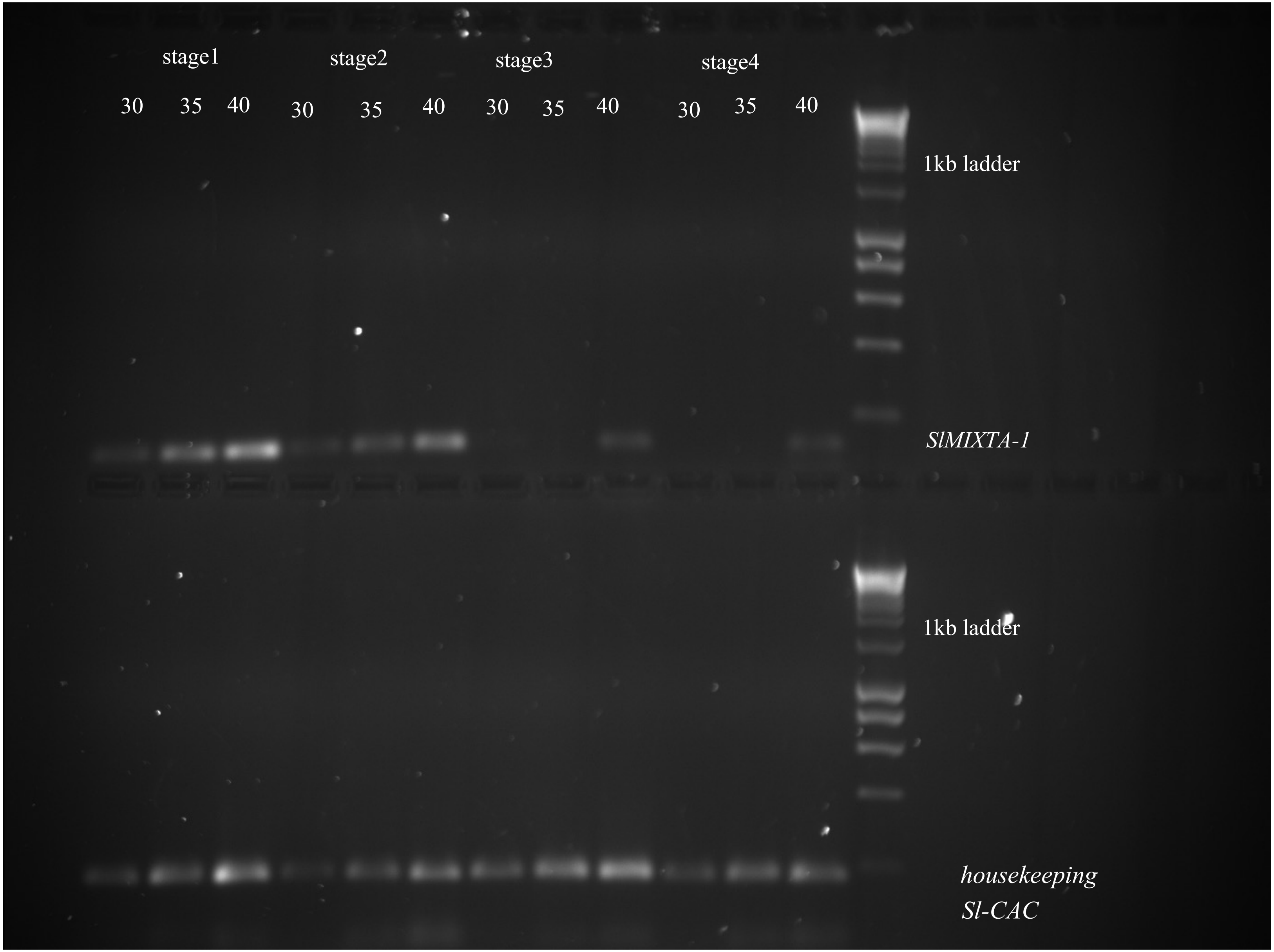

SlMIXTA-2 semiQPCR uncropped Gel image used for Fig.5

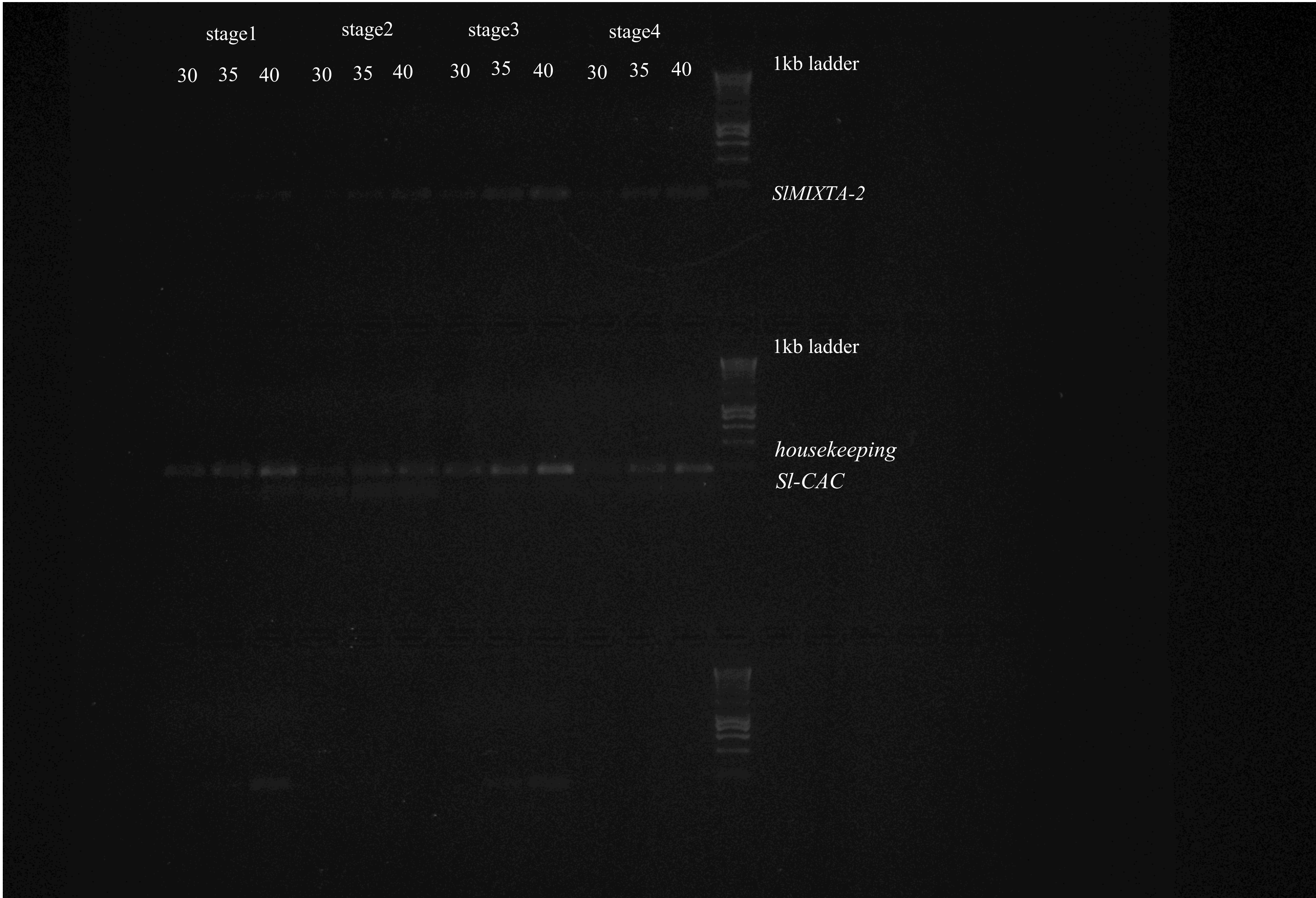

SlMIXTA-3 semiQPCR uncropped Gel image used for Fig.5

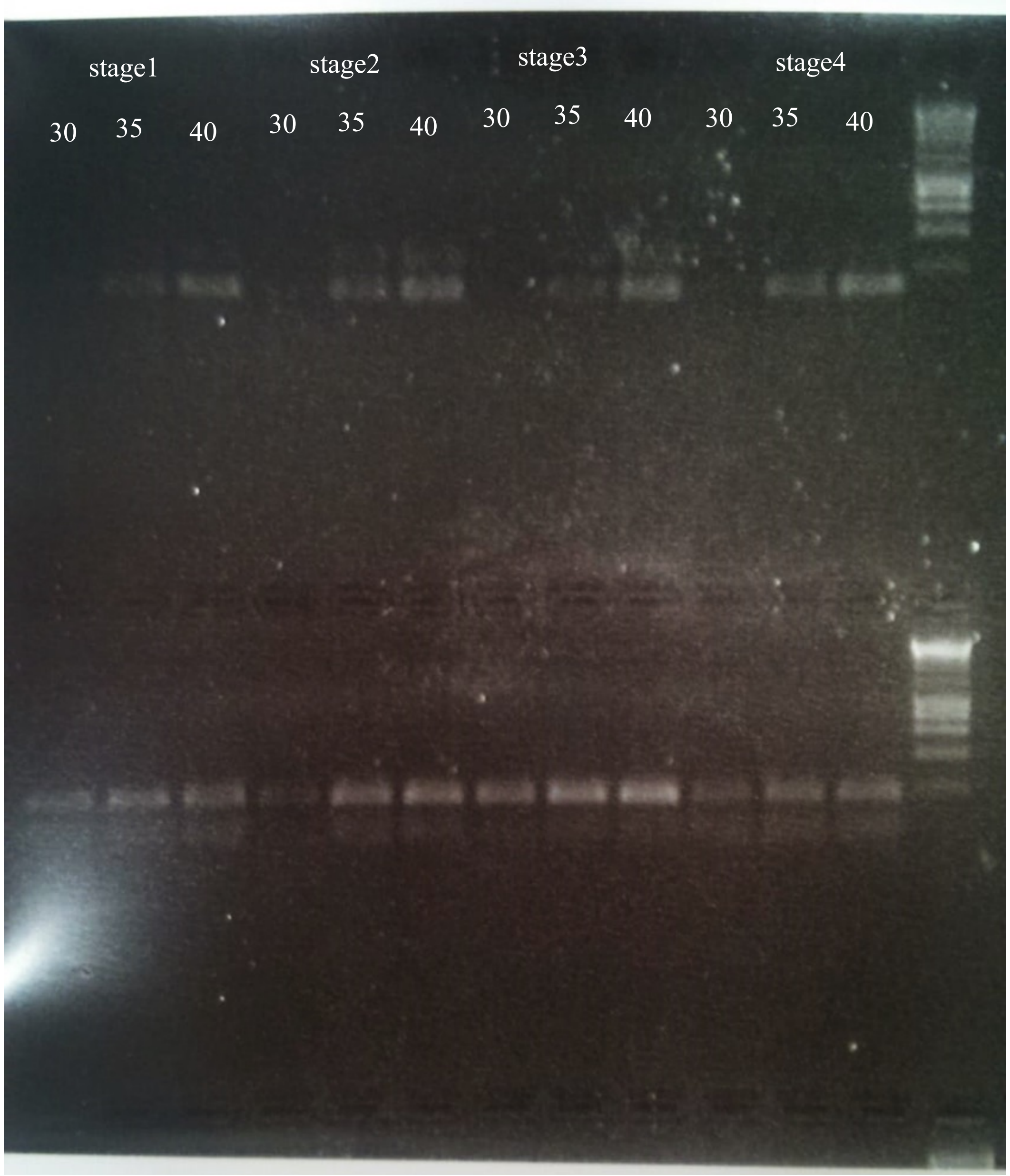

SlMIXTA-4 semiQPCR uncropped Gel image

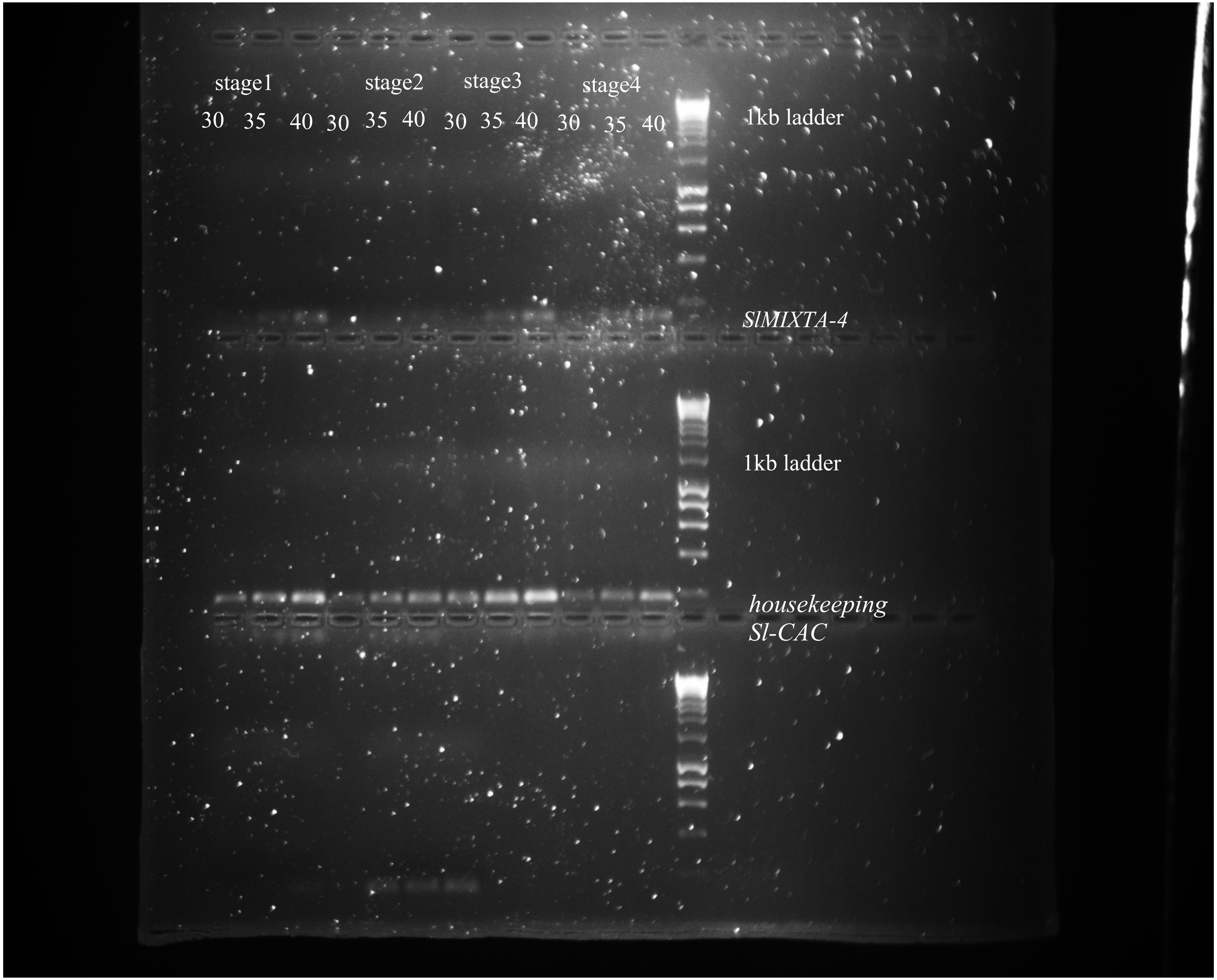

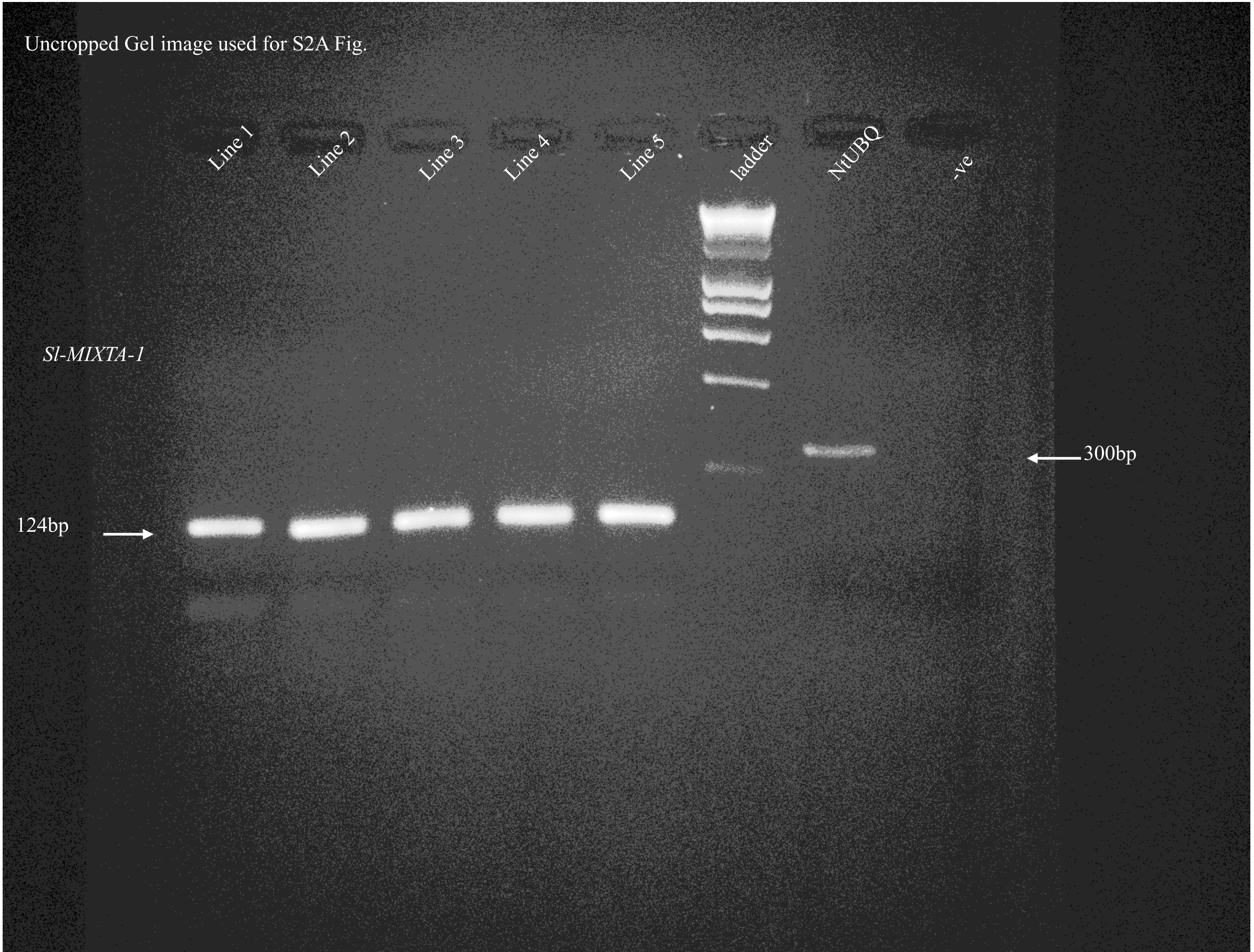

Uncropped Gel image used for S2A Fig.

Uncropped Gel image used for S2B Fig.

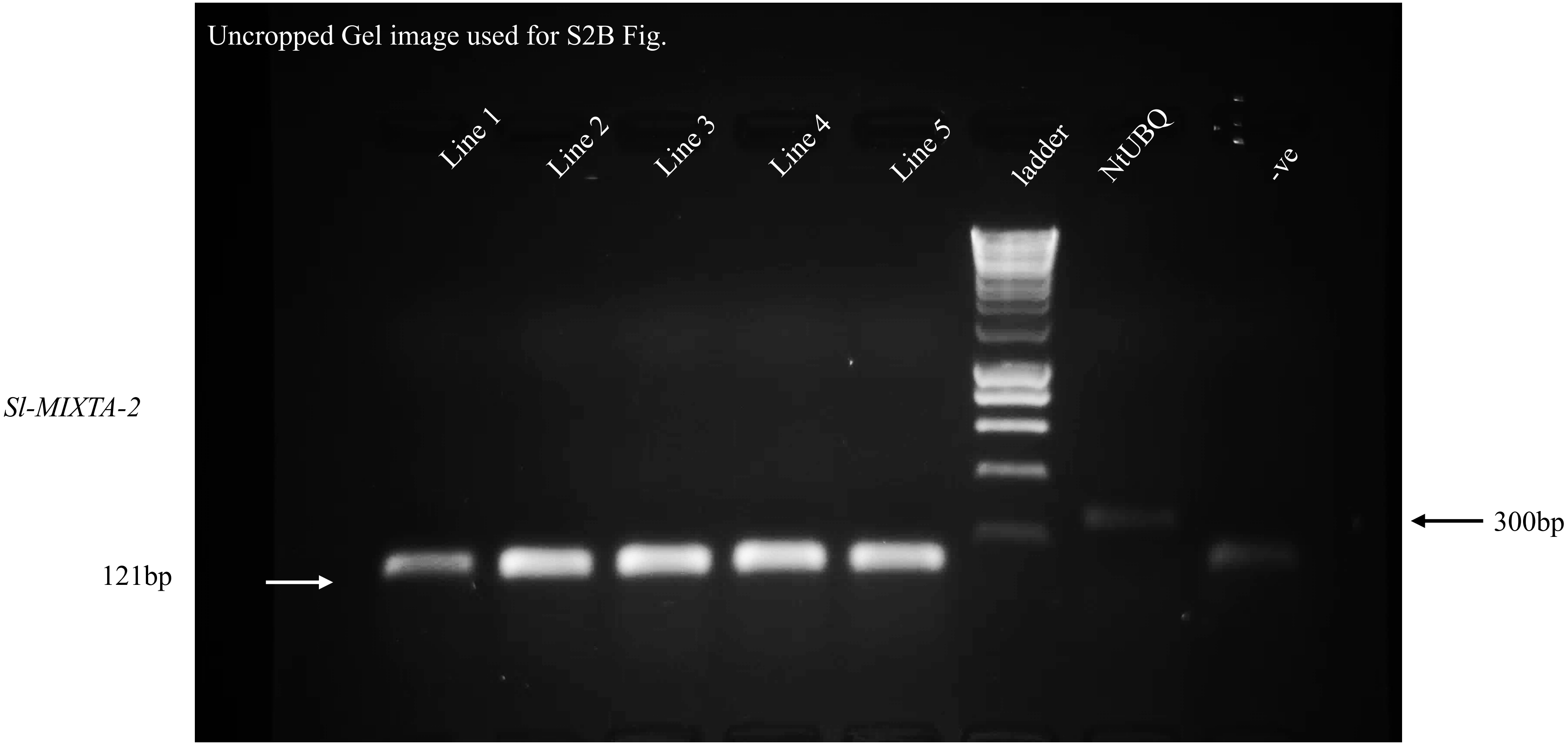

Uncropped Gel image used for S2C Fig.

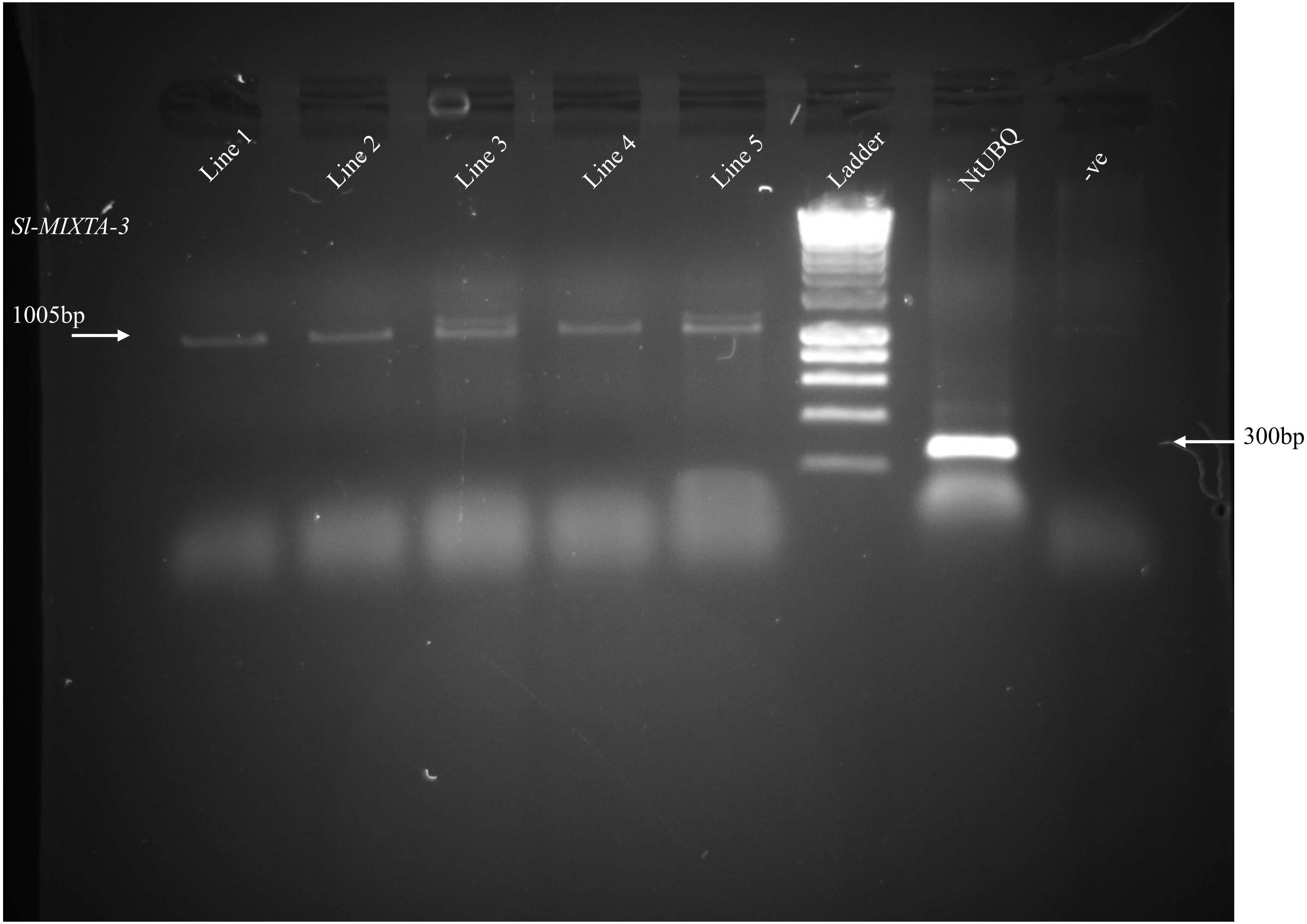

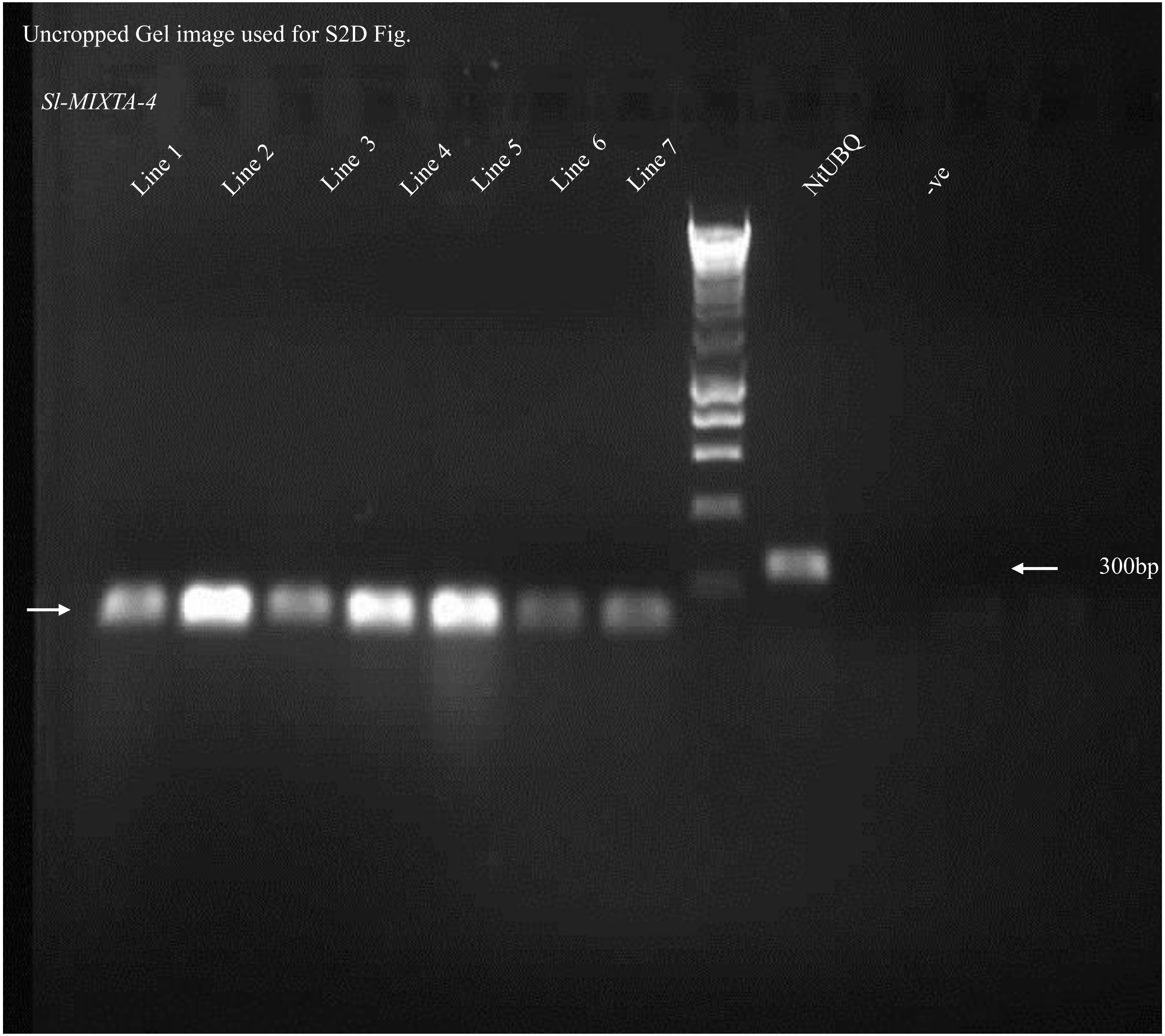
Uncropped Gel image used for S2D Fig.
Sl-MIXTA-4
Line 1
Line 2
Line 3
Line 4
Line 5
Line 6
Line 7
NtUBQ
-ve
300bp
138bp

Uncropped Gel image used for S2E Fig.

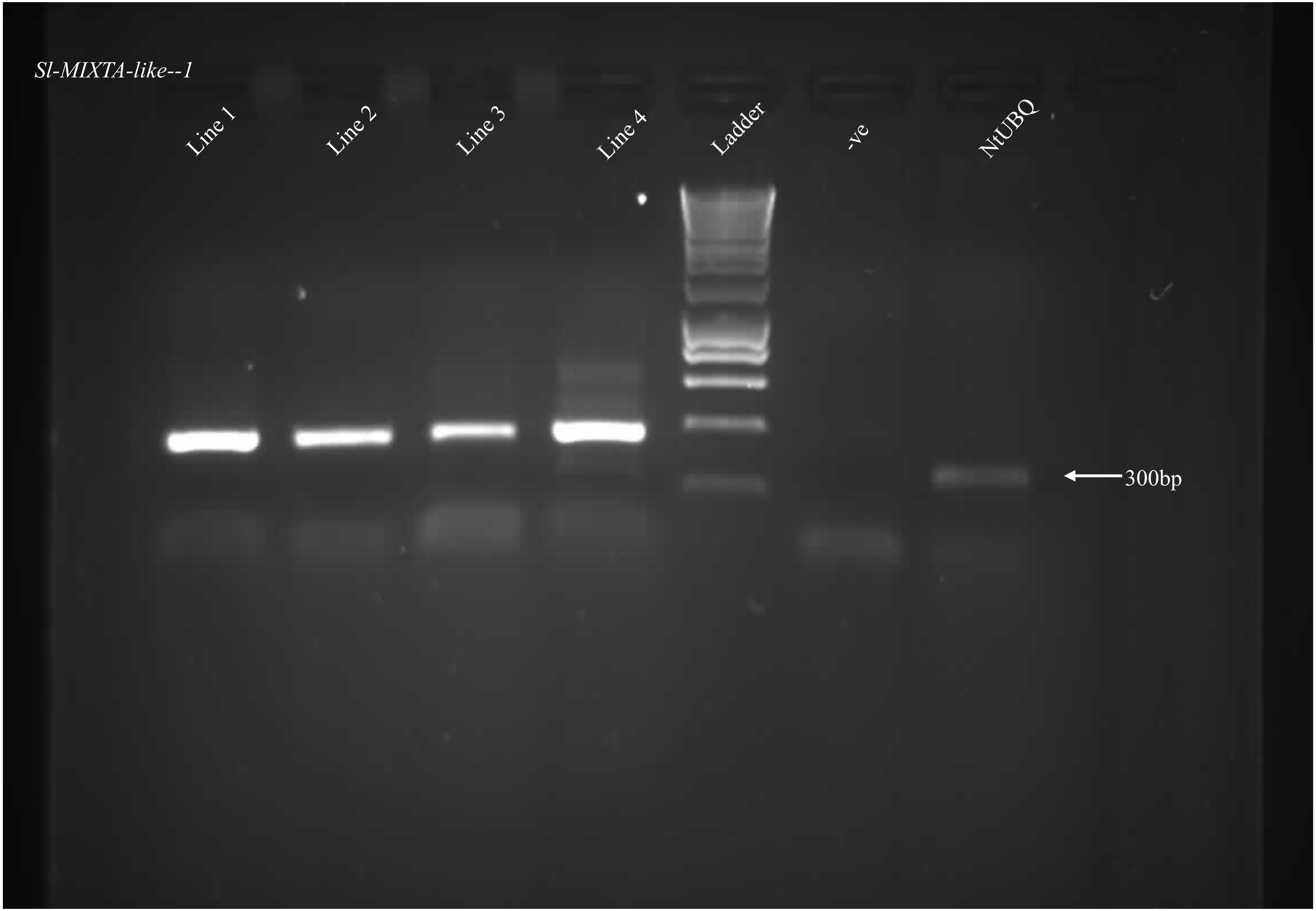

Uncropped Gel image used for S3A Fig.

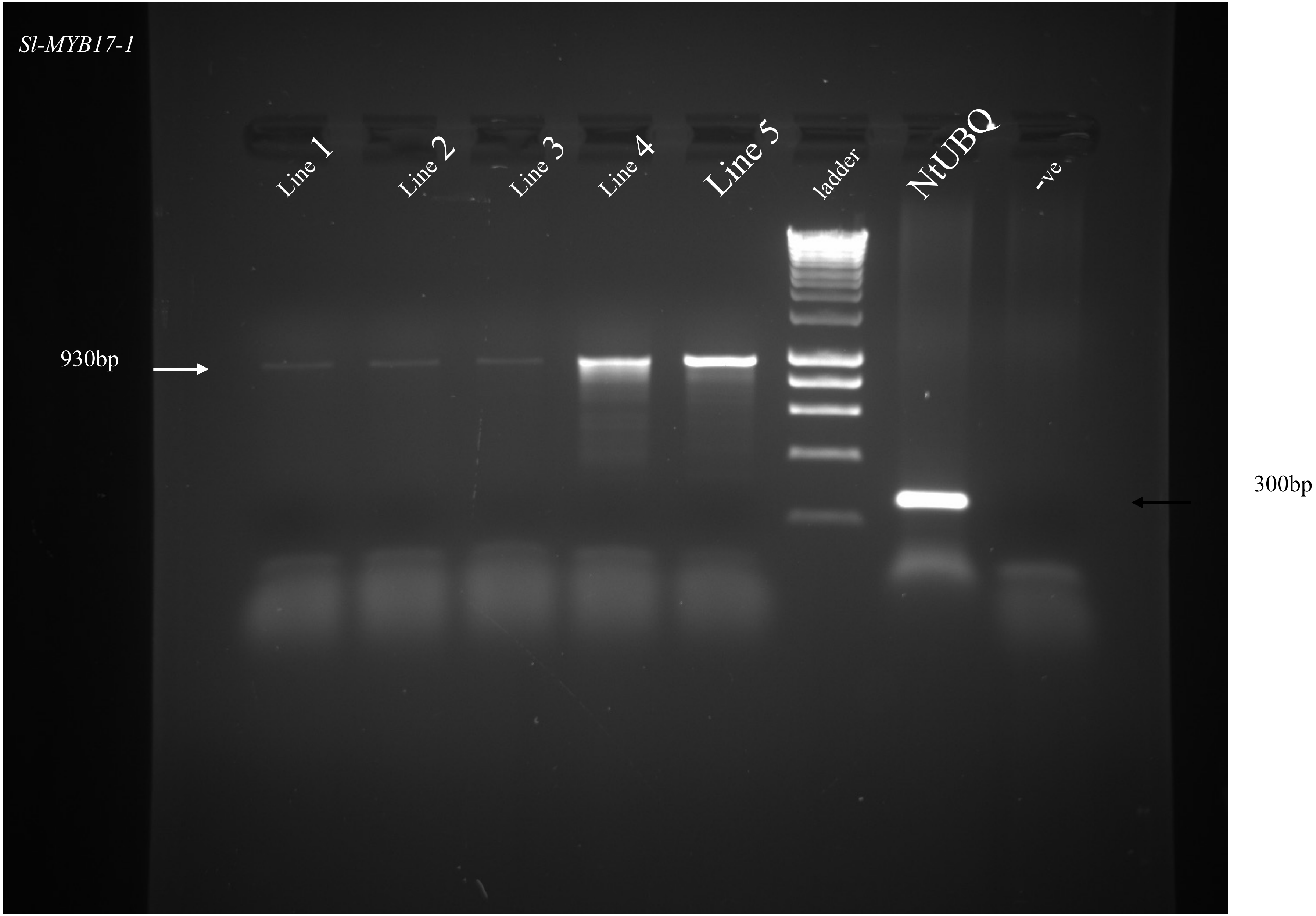

Uncropped Gel image used for S3B Fig.

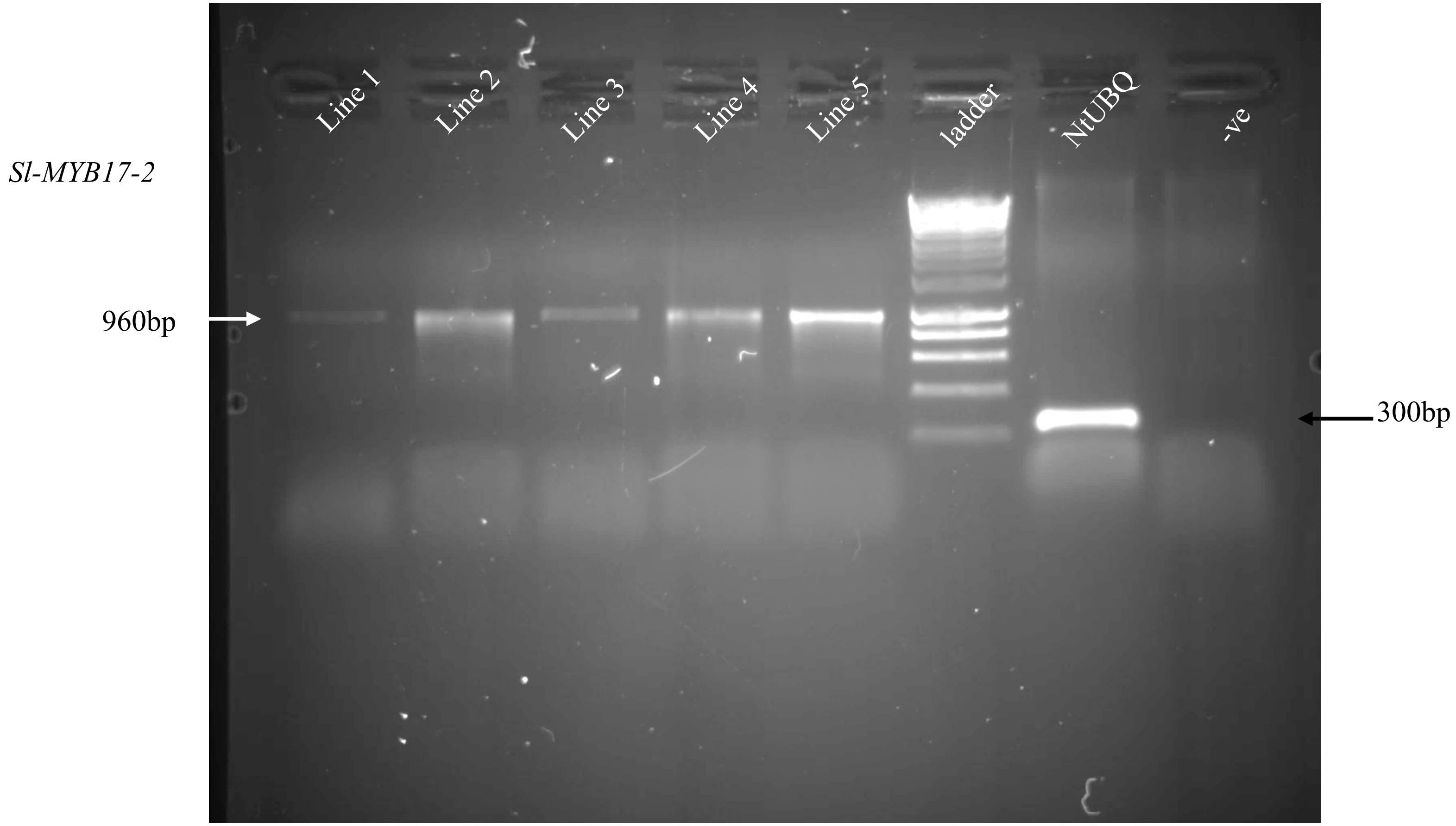

Supplement: S4 Fig — (PDF) [file pone.0295445.s004.pdf]
