# Supplementary material for: Characterisation of the R2R3 Myb subgroup 9 family of transcription factors in tomato
Source: PLoS One. 2024 Mar 26;19(3):e0295445. doi: 10.1371/journal.pone.0295445 (PMC10965086; doi:10.1371/journal.pone.0295445)
Supplement: S1 Table — (PDF) [file pone.0295445.s005.pdf]

Supplementary table 1

Primer Name	Sequence	Purpose
SlMX-1-Fw	ATGGGAAGGTTTGATAAAGAGGGA	Gene specific primers for amplification of full length coding sequence of Solyc05g007710.2.1 (S*lMIXTA-1)*
SlMX-1-Geno-Rv	CAAATAATTAGGGAAATTATTCCAATTAAT	Gene specific primer used for genotyping transgenics containing *SlMIXTA-1*
SlMX-1-Rv	TTAGTTAATACAATCAATAGGTGAATTAAC	Gene specific primers for amplification of full length coding sequence of solyc05g007710.2.1 (S*lMIXTA-1)*
SlMX-2-Fw	ATGGGAAGATCACCATGTTTAGAT	Gene specific primers for amplification of full length coding sequence of Solyc04g005600.1.1 (S*lMIXTA-2)*
SlMX-2-Rv	TTAGAGTAATGGAGATCCAATTGG	
SlMX-3-Fw	ATGGGAAGATCACCATGTTGTG	Gene specific primers for amplification of full length coding sequence of Solyc01g010910.1 (S*lMIXTA-3)*
SlMX3-Rv	TTAAAATACTGATGAACCATCAACAA	
SlMX4-Fw	ATGGGAAGATCCAAATATTGTGAT	Gene specific primers for amplification of full length coding sequence of Solyc05g007690.1 (S*lMIXTA-4)*
SlMX4-Rv	TTAAAATACTGGAGAACCAATAGG	
SlMX-like-1-Fw	ATGGGTCGATCTCCGTGTTG	Gene specific primers for amplification of full length coding sequence of Solyc02g088190.2.1 (S*lMIXTA-like-1)*
SlMX-like-1-Rv	TTAGAACATAGATGAATCAGATGGAG	
SlMyb17-1-Fw	ATGGGGAGAACACCATGTTGTG	Gene specific primers for amplification of full length coding sequence of Solyc01g094360 (S*lMYB17-1)*
SlMyb17-1-Rv	TCATGAGCTTTCACTCAAAAAGGG	
SlMyb17-2-Fw	ATGGGGAGAACACCGTGTTG	Gene specific primers for amplification of full length coding sequence of Solyc05g048830.2.1 (S*lMYB17-2)*
SlMyb17-2-Rv	TCAGAGAAAGCCAACTTCATGTTG	
CAC-Fw	CCTCCGTTGTGATGTAACTGG	Reference primers using CAC gene in tomato for use as positive control
CAC-Rv	ATTGGTGGAAAGTAACATCATCG	
SQ-SlMX-1-Fw	GCATGGCAAATAGCTAGCAC	Forward primer for semi-qRTPCR of Solyc05g007710.2.1 *SlMIXTA-1*
SQ-SlMX-1-Rv	CTCCGAGGAATTCAACGATG	Reverse primer for semi-qRTPCR of Solyc05g007710.2.1 *SlMIXTA-1*
SQ-SlMX-2-Fw	CCCTGGAAACTACTCAAGTG	Forward primer for semi-qRTPCR of Solyc04g005600.1.1 *SlMIXTA-2*
SQ-SlMX-2-Rv	ATGGAGATCCAATTGGTGAAG	Reverse primer for semi-qRTPCR of Solyc04g005600.1.1 *SlMIXTA-2*
SQ-SlMX-3-Fw	TTAAAAGCATGGCAAGGAGG	Forward primer for semi-qRTPCRof Solyc01g010910.1 *SlMIXTA-3*
SQ-SlMX-3-Fw	ATCAATTAATCCAGCCCCTG	Reverse primer for semi-qRTPCR of Solyc01g010910.1 *SlMIXTA-3*
SQ-SlMX-4-Fw	AAGATCCAAATATTGTGATGAAGAG	Forward primer for semi-qRTPCR of Solyc05g007690 *SlMIXTA-4*
SQ-SlMX-4-Rv	TTGCAGACCAGCTTTAGCAG	Reverse primer for semi-qRTPCR of Solyc05g007690 *SlMIXTA-4*
SQ-SlMX-like-1-Fw	TTGGAAGGAATTTGGGAACTC	Forward primer for semi-qRTPCR of Solyc02g088190.2.1 *SlMIXTA-like-1*
SQ-SlMX-like-1-Rv	TCTGTTGTCCATGTTGTGTC	Reverse primer for semi-qRTPCR of Solyc02g088190.2.1 *SlMIXTA-like 1*
