# Supplementary material for: Characterisation of the R2R3 Myb subgroup 9 family of transcription factors in tomato
Source: PLoS One. 2024 Mar 26;19(3):e0295445. doi: 10.1371/journal.pone.0295445 (PMC10965086; doi:10.1371/journal.pone.0295445)
Supplement: Attachment — Submitted filename: response to reviewers.pdf [file pone.0295445.s007.pdf]

Dear Reviewers,

Thank you for your time in reviewing this manuscript for submission to PLOS ONE. Please find bellow listed responses to the Reviewers' comments;

Reviewer #1:

1. The methodology overview is concise but would benefit from a brief rationale for selecting tobacco as the host for ectopic expression.

Response: The following explanation has been added into methods section: "Tobacco was selected as the host for ectopic expression because of its status as a model species for transgenic studies due to its ease of transformation and its well characterised phenotype. It has also been used for ectopic expression in many previous studies exploring the function of MYB subgroup 9 proteins. Therefore, this allows functional comparison between genes from different species."

2. While the content mentions variation in epidermal cell outgrowth, specific phenotypic data for each ectopically expressed gene would strengthen the presentation.

Response: Thank you for this helpful suggestion, we have included a table which summarises the phenotypes induced by all 7 genes in 3 major tissues.

3. Identifying SlMIXTA-2 and SlMYB17-2 as candidate regulators is promising, but clarifying the selection criteria is advisable.

Response: Additional sentences have been added to the paper's conclusion expanding on this slightly: "*SlMIXTA-2* is expressed most strongly in the leaves, and not expressed in the floral tissue. *SlMYB17-2* is the best candidate for the regulation of the anther trichome mesh. *SlMYB17-2* is expressed strongly in the floral tissue and produces a clear phenotype of epidermal cell outgrowths when ectopically expressed in tobacco."

4. Expanding on the implications of these findings for plant biology and applications would provide a broader context.

**Response: Thank you for this helpful suggestion. We have now added a new final paragraph addressing this:
"The cellular outgrowth induced by members of this transcription factor family plays important roles in various aspects of plant development and physiology, including in interactions with herbivores and pollinators and in macroscopic shaping of organs. Further analyses of the regulation of cellular outgrowth will allow greater understanding of these processes and support potential applications in improving crop plants."**

5. - Phylogenetic Analysis: While the paper mentions the use of a phylogeny from a previous study (GARL1 maximum likelihood phylogram of 220 members of the subgroup 9 R2R3 Myb genes), it would be beneficial to provide more details about the specific methodology and parameters used in generating this phylogeny, as the accuracy of subgroup assignment relies heavily on this analysis.

response: Following has been added to the relevant part of the methods section, further expanding upon the phylogenetic analysis conducted in (Brockington et al, 2013) "*ML bootstrap analysis was conducted using default parameters and 100 replicates. Five replicates for GARL1 analysis were conducted and the topology with the highest likelihood score selected. In this study the phylogenetic analysis was conducted on nucleotide alignments which were aligned previously by codon; any non-confident aligned amino acids were excluded during re-iterative preliminary analysis which explored effects of exclusion and the most robust phylogenetic trees selected from preliminary analysis.*"

6. The paper mentions the use of the sequenced tomato genome as viewed on Phytozome V12.1 and Sol Genomics Network, with the current tomato genome version SL3.0 and Annotation ITAG3.10. Given that genome annotations can evolve over time, did you encounter any discrepancies or challenges in aligning candidate genes with the reference genome annotations? How were such issues resolved?

Response: We did not encounter any difficulties. Repeated checks of the genome assemblies only ever revealed these 7 genes.

7. Clarify: Tissue-Specific Effects: Are there tissue-specific effects of these genes' expression in tobacco? Do certain tissues or organs exhibit more pronounced epidermal cell outgrowths, and is there a correlation with their native functions in Solanum lycopersicum?

Response: In response to this we have added a new table summarising these tissue specific effects for each gene.

8. clarify: Stomata Formation: The presence of stomata on some trichomes is intriguing. Could you discuss the potential implications of this observation? Is there evidence to suggest a functional role for these stomata in the context of altered epidermal structures?

Response: There is some evidence to suggest (at least in *Arabidopsis*) that the regulatory circuits of stomata, trichomes and other cell type patterning in various tissues are interlinked. However the nature of this developmental link is not fully understood (Simon et al, Plant Direct 2020; Torrii Ann Bot 2021). The presence of stomata on trichomes is likely a consequence of the over-expression of these trichome related genes interacting with the regulatory circuit of stomata formation/patterning. There is probably no functional role for these stomata; or at least none that we have evidence for. It would be fascinating if future studies uncovered the link between stomata and trichome development and cell type pattern formation.

9. Reproductive Consequences: The inability of anthers to dehisce in some transgenic lines is notable. Have you explored the reproductive consequences of this phenotype? Does it impact pollination or seed production in tobacco plants, and could similar effects be expected in Solanum lycopersicum?

Response: In the paper it is already mentioned that these plants had to be hand pollinated. We have expanded this sentence to provide greater clarity as to the reproductive consequences, as follows: "*Lines ectopically expressing SlMIXTA-1 had anthers which did not dehisce even when flowers matured. This phenotype had reproductive consequences for these transgenic tobacco plants; they had to be pollinated by hand in order to produce seed.*"
Therefore the reproductive consequences of this phenotype are apparent (and quite stark.) We note that ectopic expression of related genes from *Antirrhinum majus* also prevented tobacco anther dehiscence (Glover et al 1998, Development) and we believe that the phenomenon is a result of conversion of those cells usually responsible for anther dehiscence into conical or trichome cell fates.
The anthers of tobacco and tomato are very different: tobacco anthers dehisce by opening up to release pollen, whilst tomato anthers do not. Tomato (along with all members of the genus *Solanum*) has poricidal anthers: pollen contained within a tube-like anther is released from pores at the tip in response to buzz pollination. As a result of this difference of anther type and pollination mechanism such outgrowth on the anther surface would not have the same effect in tomato: there is no dehiscence by opening to be prevented. In fact, cell outgrowths on the surface of tomato anthers are likely possible for this very reason: they do not need to dehisce so there is no interference in the anthers' ability to release pollen. It is possible that such outgrowths are in fact beneficial for pollinator grip during buzz pollination (like conical cells on flower petals).

10. Cellular Changes in Ovary: For the elongated and multi-lobed trichome-like outgrowths on the ovary surface, have you conducted any cellular or histological studies to examine the structural changes at the cellular level? Are there indications of altered cell expansion or differentiation patterns?

Response: We have not conducted any studies on the ovary surface, beyond the SEMs shown here. Future studies will analyse the ectopic cell outgrowths on each tissue and arising from each gene systematically, but that is beyond the scope of this manuscript.

Reviewer #2

11. Many words are not same format, especially figures.

Response: Thank you for noting this. It has now been fixed.

12. Table is attached by image not by Table tool.

Response: Thank you for this feedback, the appropriate tool has now been used for the table.

13. I already mentioned all my commendations in file and authors need to recheck them.

Response: Thank you for your feedback; all your proposed changes have been implemented.

14. Fig. 1A and 1B should be consolidated into one file. Fig. 1 can be just one file, comprising panels A to B, rather than one file for panels A and one file for panel B.

Response: These have now been consolidated into a single file for submission.

Dear Nguyen Hoai Nguyen, Academic Editor at PLOS ONE,

Thank you for your time in reviewing this paper. I have addressed your notes on the additional journal requirements as follows:

Journal Requirements:
When submitting your revision, we need you to address these additional requirements.

1. Please ensure that your manuscript meets PLOS ONE's style requirements, including those for file naming. The PLOS ONE style templates can be found at https://journals.plos.org/plosone/s/file?id=wjVg/PLOSOne_formatting_sample_main_body.pdf and https://journals.plos.org/plosone/s/file?id=ba62/PLOSOne_formatting_sample_title_authors_affiliations.pdf
   We have checked and formatted to adhere to these style requirements.

2. Please note that in order to use the direct billing option the corresponding author must be affiliated with the chosen institute. Please either amend your manuscript to change the affiliation or corresponding author, or email us at plosone@plos.org with a request to remove this option.
   We have changed the corresponding author to Beverley J. Glover to adhere to this point.

3. PLOS ONE now requires that authors provide the original uncropped and unadjusted images underlying all blot or gel results reported in a submission's figures or Supporting Information files. This policy and the journal's other requirements for blot/gel reporting and figure preparation are described in detail at https://journals.plos.org/plosone/s/figures#loc-blot-and-gel-reporting-requirements and https://journals.plos.org/plosone/s/figures#loc-preparing-figures-from-image-files.

The original gel images will be uploaded to the University of Cambridge Apollo repository and linked to the paper. They will be in that repository and a link will be provided once the manuscript is accepted.

4. When you submit your revised manuscript, please ensure that your figures adhere fully to these guidelines and provide the original underlying images for all blot or gel data reported in your submission. See the following link for instructions on providing the original image data: https://journals.plos.org/plosone/s/figures#loc-original-images-for-blots-and-gels.
Figures have been checked, and figure 1A and 1B combined into a single Fig1. All figures were passed through the PACE tool for resizing and resolution.

5. In your cover letter, please note whether your blot/gel image data are in Supporting Information or posted at a public data repository, provide the repository URL if relevant, and provide specific details as to which raw blot/gel images, if any, are not available. Email us at plosone@plos.org if you have any questions.
The original gel images will be uploaded to the University of Cambridge Apollo repository and linked to the paper. They will be in that repository and a link will be provided once the manuscript is accepted. Sentence explaining this has been added to the cover letter.

6. Please review your reference list to ensure that it is complete and correct. If you have cited papers that have been retracted, please include the rationale for doing so in the manuscript text, or remove these references and replace them with relevant current references. Any changes to the reference list should be mentioned in the rebuttal letter that accompanies your revised manuscript. If you need to cite a retracted article, indicate the article's retracted status in the References list and also include a citation and full reference for the retraction notice.
Reference list has been checked, and ensured to be complete and correct to the best of our knowledge at this time. Citation style has been altered to include square brackets instead of round brackets as per the journals guidelines.
